# Causal Interactions in Human Amygdala Cortical Networks across the Lifespan

**DOI:** 10.1038/s41598-019-42361-0

**Published:** 2019-04-11

**Authors:** Yuhao Jiang, Yin Tian, Zhongyan Wang

**Affiliations:** Bio-information College, ChongQing University of Posts and Telecommunications, ChongQing, 400065 China

## Abstract

There is growing evidence that the amygdala serves as the base for dealing with complex human social communication and emotion. Although amygdalar networks plays a central role in these functions, causality connectivity during the human lifespan between amygdalar subregions and their corresponding perception network (PerN), affiliation network (AffN) and aversion network (AveN) remain largely unclear. Granger causal analysis (GCA), an approach to assess directed functional interactions from time series data, was utilized to investigated effective connectivity between amygdalar subregions and their related networks as a function of age to reveal the maturation and degradation of neural circuits during development and ageing in the present study. For each human resting functional magnetic resonance imaging (fMRI) dataset, the amygdala was divided into three subareas, namely ventrolateral amygdala (VLA), medial amygdala (MedA) and dorsal amygdala (DorA), by using resting-state functional connectivity, from which the corresponding networks (PerN, AffN and AveN) were extracted. Subsequently, the GC interaction of the three amygdalar subregions and their associated networks during life were explored with a generalised linear model (GLM). We found that three causality flows significantly varied with age: the GC of VLA → PerN showed an inverted U-shaped trend with ageing; the GC of MedA→ AffN had a U-shaped trend with ageing; and the GC of DorA→ AveN decreased with ageing. Moreover, during ageing, the above GCs were significantly correlated with Social Responsiveness Scale (SRS) and State-Trait Anxiety Inventory (STAI) scores. In short, PerN, AffN and AveN associated with the amygdalar subregions separately presented different causality connectivity changes with ageing. These findings provide a strong constituent framework for normal and neurological diseases associated with social disorders to analyse the neural basis of social behaviour during life.

## Introduction

Emotional characteristics differ greatly over the human lifespan (from development to ageing). In recent years, social perception, affiliation and aversion have attracted significant attention due to their fundamental role in human interaction dynamics. In previous research, when judging social cognitive developmental trajectories, children and older adults obtained low accuracy rating scores, while adolescents and young adults received high scores. Compared to adolescents and children, adult emotion was more stable when faced with environmental stimuli^1^. Moreover, the ability to process emotional information was stable in adults, but emotional control indicated a positive emotional bias, where the person tended to weaken negative emotion and enhance positive emotion^2,3^. Although studies have provided some interesting insights, a full explanation for this phenomenon has yet to be established. Notably, the above discrepancy could result from lower ecological validity or cue utilisation validity in children and older adults than in adolescents and young adults^4^.

In humans, the amygdala plays a key role in emotional control. For instance, the functional connectivity density of an amygdala-based network increases with age^5^, in contrast with decreases in the nodes of other networks involved in anxiety. Indeed, the connectivity between the amygdala and attentional networks increases linearly with age^6,7^. Other studies have suggested that the amygdala is a key hub in social cognitive and emotional systems, and it is involved in face perception together with fusiform gyrus^8,9^. Additionally, the amygdala encodes and affects social emotional stimuli when processing faces and subjective judgments of facial expressions^10–12^. As such, the amygdala could contribute to the integrative processing of social information that underlies the awareness of other individual’s affective experiences in complex social perception.

Previous research about lifespan emotional changes focused on the amygdala as a whole^13^, but some studies suggested that the amygdalar role in cognition, disapproval and sympathy might be separable. For example, Bickart and colleagues divided the amygdala into three subregions, namely the ventrolateral amygdala (VLA), medial amygdala (MedA) and dorsal amygdala (DorA), and indicated that these subregions exert important roles in perception, affiliation and aversion, respectively^7^. The perception network (PerN) is responsible for social perceptual abilities, the affiliation network (AffN) is related to prosocial behaviours and the aversion network (AveN) contributes to antipathetic processes. Further, the amygdala cooperates with many other brain regions to process social emotional information at the network level^14–17^. For example, the PerN is involved with the lateral orbitofrontal cortex (lOFC), fusiform gyrus (FFA), rostral superior temporal sulcus (rSTS), ventromedial temporal cortex, temporal pole and subgenual anterior cingulate cortex (ACC); the AffN is associated with the ventromedial prefrontal cortex (vmPFC), ventral medial striatum of the nucleus accumbens, ventromedial hypothalamus, adjoining subgenual and rACC, dorsomedial temporal pole and medial temporal lobe; and the AveN refers to caudal ACC, ventrolateral striatum, anterior insula, somatosensory operculum, caudolateral hypothalamus, thalamus and brainstem^7,18–27^.

Interestingly, connectivity patterns represent various trajectories during human ageing. Short- and long-distance connectivity between brain regions shows an upright and inverted U-shaped trajectory, respectively, between brain regions^28^. The connectivity within/between functional networks separately presented inverted/upright U-shaped trends^29,30^. Moreover, functional flexibility shows various changes in trajectory with ageing. The lateral frontal and parietal lobe exhibit an inverted and upright U-shaped trend, respectively^31^.

Critically, early studies explored age-related connections between the amygdala and other brain networks largely in terms of examining the strength of functional connectivity. For example, enhanced amygdalar activity is related to stronger activation of visual-, attentional- and emotional-related neural networks^32–34^. Since the amygdala plays a modulatory role within this network, where it can enhance neural responses in visual areas and perceptual ability for affect-laden stimuli^35,36^, increased amygdalar activity is linked to increased visual acuity^37^ and greater visual cortex activation, including area V1^38^. However, the usual methods, such as time series correlation analysis on region of interests (ROIs), resting-state connectivity maps using independent component analysis (ICA) and large-scale functional connectivity networks, do not always explicitly explain the functional interaction between brain regions. Besides, simultaneous activation of two anatomically disconnected neural circuits by an external stimulus does not reveal potential effective connectivity between them. Importantly, effective connectivity refers to the influence that one neural circuit exerts over another and indicates the information flow direction. More clearly, functional connectivity refers to the factor of simultaneity. In contrast, GCA is one of analysis models for effective connectivity, and it emphasises causality between brain areas^39^. In practice, as reported in early research, the conclusion that the amygdala exerts an important role in the PerN was derived from their correlation, but the information flow direction between them is still unclear. Thus, in this study we proposed a novel approach to examine the flows of Granger causality (GC) between the amygdalar subregions (VLA, MedA or DorA) and their corresponding networks (PerN, AffN or AveN) during the entire human lifespan based on an age-related generalised linear model (GLM). We investigated the correlation between effective connectivity changes and human behaviour alteration at different ages and aimed to reveal the neurally mediated mechanisms that involve the amygdala and their networks during the human lifespan.

## Results

### Identification of amygdalar subregions

**Figure 1:**
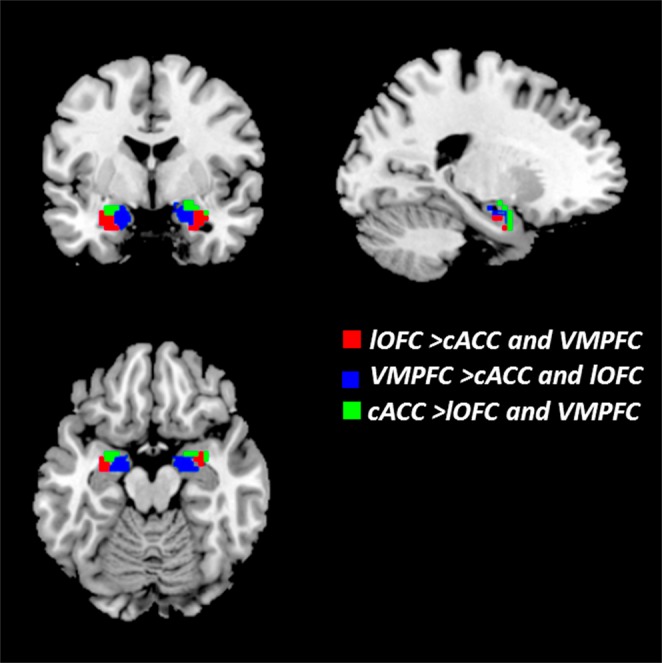
Three connectionally defined subregions of the amygdala. Three voxel clusters demonstrate strongest functional connectivity with lOFC, VMPFC and cACC, respectively. The red brain region represents VLA, lOFC > cACC and VMPFC; the blue brain region represents MedA, VMPFC > cACC and lOFC; the green brain region represents DorA, cACC > lOFC and VMPFC.

Using the three cortical ROIs (lOFC, VMPFC and cACC) as seed regions, the amygdalar subregions were defined by connectional analysis, as shown in Fig. 1. According to Harvard-Oxford 50% probability maps of the entire amygdala, we divided the amygdala into three subregions that encompassed the social-perception-relevant VLA, the social-affiliation-relevant MedA and the social-aversion-related DorA. The coordinates of peak nodes for identifying amygdalar subregions are shown in Table 1.

**Table 1:** Identification of amygdala nuclei.

Anatomical	Hemisphere	Cluster voxels	MNI(x,y,z)
Ventrolateral amygdala	L	36	(−27, −3, −21)
Ventrolateral amygdala	R	45	(33, 0, −21)
Medial amygdala	L	20	(−15, −8, −18)
Medial amygdala	R	26	(18, −9, −18)
Dorsal amygdala	L	18	(−24, −6, −12)
Dorsal amygdala	R	17	(18, 0, −18)

### Identification of amygdalar subregion-based social networks

**Figure 2:**
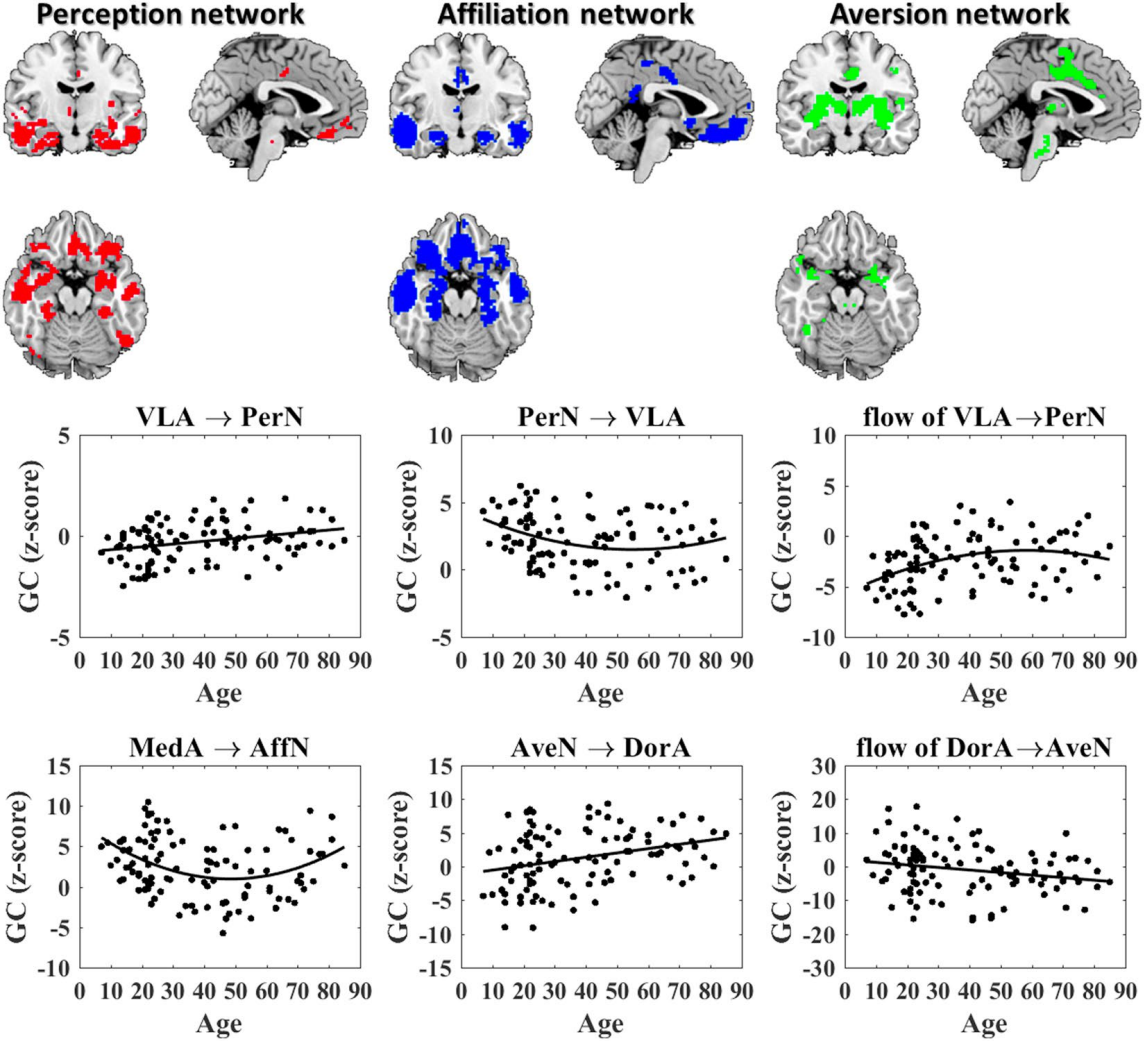
The trends of Granger causality between amygdala subregions and associated networks with age.

The spatial patterns for social networks (PerN, AffN and AveN) were depicted via the binary intersection maps of amygdalar subregions (VLA, MedA and DorA) and corticolimbic regions (lOFC, VMPFC and cACC), as shown in Fig. 2 and Table 2.

**Table 2:** Distribution of brain regions associated with the amygdala nuclei based social networks.

Anatomical	Hemisphere	Cluster voxels	MNI(x, y, z)	T
***PerN***				
VLA	L	55	(−27, −3, −18)	57.36
VLA	R	35	(26, −3, −21)	58.27
medial OFC	L	110	(−6, 48, −12)	14.22
medial OFC	R	87	(6, 48, −12)	15.06
lOFC	L	293	(−31, 30, −18)	49.62
lOFC	R	297	(36, 32, −18)	50.44
FFA	L	288	(−31, −33, −20)	16.10
FFA	R	350	(35, −34, −21)	18.44
rSTS	L	374	(−52, −4, 21)	13.66
ventralmedial temporal cortex	L	154	(−12, −27, 0)	14.65
ventralmedial temporal cortex	R	148	(12, −22, 6)	14.68
temporal pole	L	160	(−51, 9, −18)	17.09
temporal pole	R	157	(49, 9, −16)	18.18
subgenual ACC	R	161	(−6, 0, 30)	13.38
***AffN***				
MedA	L	86	(−15, −3, −18)	42.99
MedA	R	69	(14, −3, −20)	30.76
VMPFC	L/R	203	(0, 32, −13)	43.25
ventral medial striatum	L	83	(−11, 12, −9)	8.10
ventral medial striatum	R	174	(9, 12, −11)	10.42
ventral medial hypothalamus	L	25	(3, −5, −16)	9.35
ACC	L	121	(−3, 45, 0)	29.72
dorsomedial temporal pole	L	40	(−24, −14, −22)	12.11
dorsomedial temporal pole	R	37	(33, −15, −21)	10.55
medial temporal lobe	L	420	(−54, −60, 3)	10.39
medial temporal lobe	R	446	(50, −61, 3)	7.44
***AveN***				
DorA	L	45	(−18, −3, −12)	48.85
DorA	R	24	(21, −3, −15)	58.38
cACC	L/R	247	(0, 15, 33)	45.07
anterior insula	L	178	(−33, 9, 3)	31.27
anterior insula	R	147	(33, 12, −16)	13.87
somatosensory operculum	L	337	(−6, −6, 63)	20.66
somatosensory operculum	R	423	(3, −3, 63)	11.70
ventrolateral striatum	L	127	(−27, −3, 12)	18.66
ventrolateral striatum	R	135	(27, 3, 9)	17.29
caudolateral hypothalamus	R	40	(3, −6, −12)	12.58
thalamus	R	83	(3, −15, 3)	17.84
brainstem	L	239	(−6, −24, −33)	10.58

### Statistical results

#### One sample t-test results

One-sample *t*-test analysis revealed significant GC between the VLA and PerN, MedA and AffN and DorA with AveN. We also found significant GC net flow (inflow minus outflow from the network to the amygdala) from amygdalar subregions to the associated social network (p < 0.05, false discovery rate [FDR] corrected).

#### Age-related causal interaction analysis results

We investigated the changes in the causal influences from a lifespan perspective using an age-relevant GLM with linear and quadratic age terms as the predictive factor. The patterns that described the GC trend between amygdalar subregions and associated networks are shown in Fig. 2. For GC from VLA to PerN, the linear term was found to be significant (t = 3.16, p = 0.002, *R*^2^/**R** = 0.30), and the GC from VLA to PerN increased linearly with age. The GC from the PerN to VLA had a significant quadratic age term (t = 2.03, p = 0.004, *R*^2^/**R** = 0.09) and exhibited U-shaped trajectory with ageing. The net flow from VLA to PerN also exhibited a significant quadratic age effect (t = −2.10, p = 0.004, *R*^2^/**R** = 0.13) and demonstrated an age-related Inverted-U trend. For causal interaction between AffN and MedA, onlyGC from the MedA to AffN demonstrated a U-shaped trend with a significant quadratic age term (t = 3.38, p = 0.001, *R*^2^/**R** = 0.12). Neither of the GC from AffN to MedA nor the net flow from MedA to AffN showed a significant correlation with age. GLM analysis revealed that the GC from DorA to AveN was not statistically significantly correlated with age. However, the GC from AveN to DorA increased linearly as age increased (t = 3.33, p = 0.001, *R*^2^/**R** = 0.31), while the net flow from DorA to AveN decreased linearly with age (t = −2.27, p = 0.03, *R*^2^/**R** = −0.22). The statistical regression coefficients appear in Table 3. Moreover, all Granger causal interactions between the amygdala subregions (VLA, MedA, and DorA) and their corresponding networks (PerN, AffN and AveN) were not correlated to gender.

**Table 3:** Statistical parameters of age-related causal connectivity.

GC	T	p	R^2^/R	Fitting curve
VLA → PerN	3.16	0.002	**0.30**	y = 0.0139 *age* − 0.8206
PerN → VLA	2.03	0.04	0.09	y = 0.0010 *age*^2^ − 0.10833 *age* + 4.4730
VLA → PerN-PerN → VLA	−2.10	0.04	0.13	y = −0.0013 *age*^2^+0.1475 *age*−5.7180
MedA → AffN	3.38	0.001	0.12	y = 0.0030 *age*^2^ − 0.28812 *age* + 7.9426
AveN → DorA	3.33	0.001	**0.31**	y = 0.0638 *age* − 1.1736
DorA → AveN-AveN → DorA	−2.27	0.03	**−0.22**	*y* = −0.0756 *age* + 1.9887

Note: R^2^ in R^2^/R represents the curve fitting degree of the age-related quadratic fitting curve, expressed in black, R represents the correlation between GC and age.

#### Behavioural scale correlation analysis

**Figure 3:**
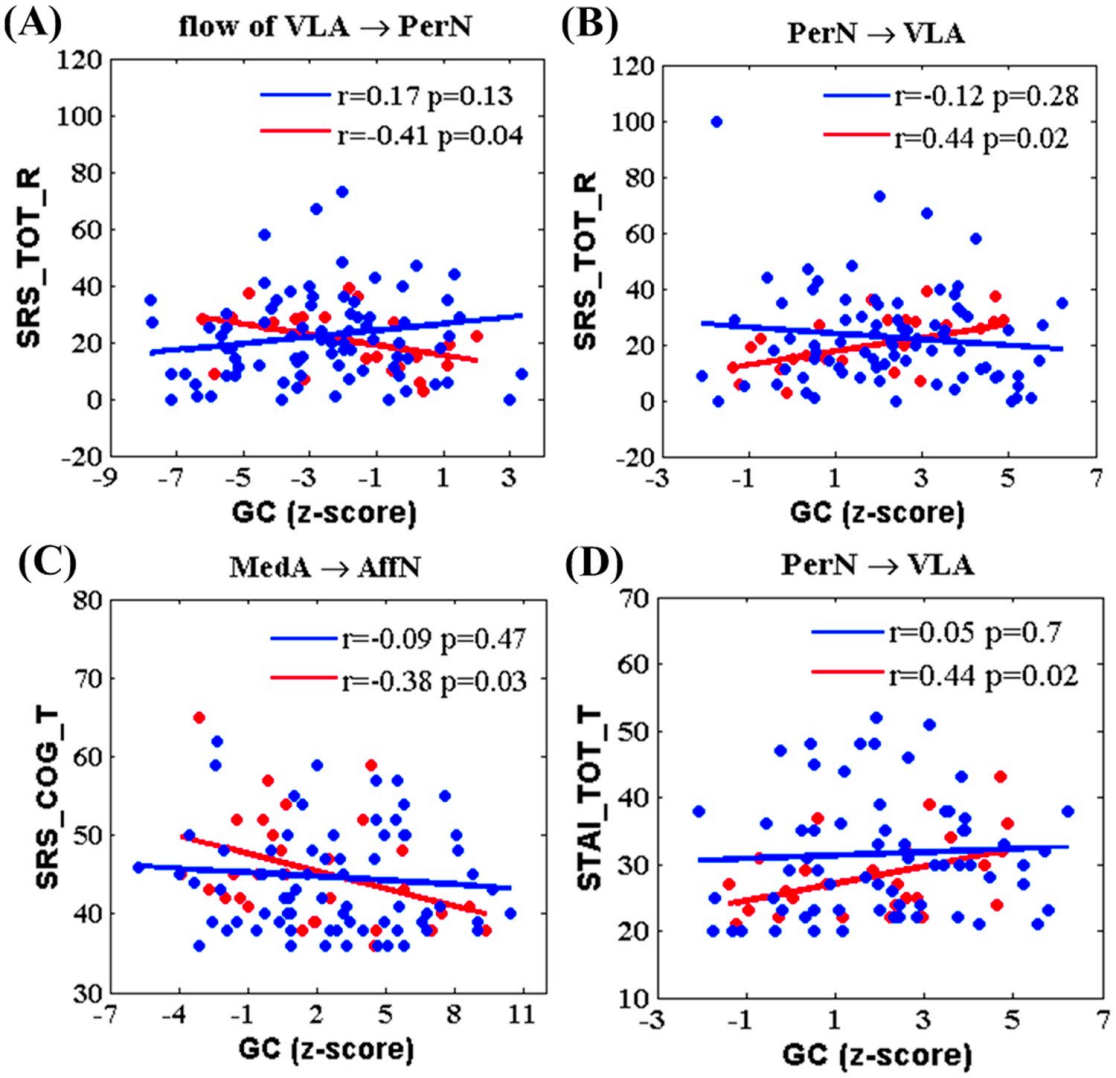
The correlation between Granger causality and behavioral scales. (**A**) Scatter plots demonstrate the relationship between GC net flow from VLA to PerN and Social Responsiveness Scale score. (**B**) Scatter plots demonstrate the relationship between GC of PerN to VLA and Social Responsiveness Scale score. (**C**) Scatter plots demonstrate the correlation between GC of MedA to AffN and Social Responsiveness Scale score. (**D**) Scatter plots demonstrate the correlation between GC and State Trait Anxiety Inventory. SRS_TOT_R represents SRS score, SRS_COG_T represents the social cognitive scale, and STAI_TOT_T represents State Trait Anxiety Inventory. The blue line and points indicates the development process, and red line and points indicates the aging process.

For the entire lifespan, there was no statistically significant correlation between GC values and Behavioral scale test results, including Social Responsiveness Scale (SRS) and State-Trait Anxiety Inventory (STAI). However, there were significant correlations between the age-dependent quadratic fitting curve of GC values and the behavioural scales (SRS and STAI) during ageing (>50 years), as shown in Fig. 3. The blue line and points represent development, while the red line and points represent ageing. The GC information flow from the VLA to PerN was negatively correlated with SRS score(r = −0.41, p = 0.04; Fig. 3A) during ageing. GC from PerN to VLA was positively correlated with SRS scores during ageing (r = −0.44, p = 0.02; Fig. 3B). GC from MedA to AffN was negatively correlated with SRS cognitive scores (r = −0.38, p = 0.03; Fig. 3C). Additionally, GC from PerN to VLA was positively correlated with STAI scores during the ageing (r = 0.44, p = 0.02; Fig. 3D). There were no statistically significant correlations between GC values and behavioural scales during development (<50 years; Fig. 3).

## Discussion

In this work, we uncovered GC trends between specific amygdalar nuclei (VLA, MedA or DorA) and their corresponding networks during the entire human lifespan using Granger causal analysis (GCA)-based on a GLM, and explored the mechanisms of different amygdalar nuclei in specific control modes during development and ageing. Further, a continuous age-relevant NKI-RS database provided a potential neural basis for better elucidating the perception, affiliation and aversion networks, and offered biomarkers for these social-network-related disorders.

The NKI-RS dataset employed in this study was also utilised in several previous studies. For instance, this dataset was used to explore functional connectivity patterns of the amygdala and visual cortex in healthy adults but precluded the influence of age^40,41^. He *et al*. investigated anxiety-relevant variations in the intrinsic functional connectivity between the amygdala and multiple cortical networks across adulthood^42^. Moreover, Yang *et al*. studied the correlation between brain function and structure by using data mining^43^. However, because each amygdalar subregion is responsible for a specific emotional function, the correlation between effective connectivity and age would be better understood in terms of segmentation.

Interestingly, Zhao *et al*. found age-related inverted U-shaped trends in network properties (network strength, cost, topological efficiency and robustness) across the human lifespan^30^. Uddin *et al*. selected the developmental and adulthood portion of the NKI-RS sample to verify stronger functional hub coupling in adults compared to children^44^. Similarly, Yang *et al*. used this dataset to show various age-related trajectories of functional network connectivity based upon independent component analysis^45^. Zuo *et al*. discovered that functional homogeneity in the human cortex might be determined by anatomical, developmental and neurocognitive factors^46^. Yin *et al*. found dissociable variations in frontal and parietal cortices in inherent functional flexibility across the entire human lifespan^31^. Cao *et al*. reported a linear decrease in the functional connectome and an inverted-U shaped lifespan trend for rich club structure^28^. Although these studies explored age-related correlation between the overall topological properties of the brain across the human lifespan, the characteristics of emotional functions during different stages (development, maturation and ageing) remains largely unclear.

Concretely, we found an increased Granger causal influence from the VLA to PerN with ageing, and the age-related GC from PerN to VLA showed a U-shaped trajectory. Further, the causal influence net flow degree from VLA to PerN (VLA → PerN - PerN → VLA) demonstrated an inverted U-shaped trend with ageing. In contrast, GC values from the MedA to AffN showed a U-shaped trend with ageing, and the GC values from AveN to DorA increased as age increased, while the net flow from DorA to AveN (DorA → AveN - AveN → DorA) decreased with ageing (Fig. 2).

### Information flow between the VLA and PerN

A growing body of work indicates that resting-state intrinsic connectivity reflects functional properties of the brain that relate to individual differences for a variety of abilities and behaviours^47–51^. The social PerN network of the human brain would change as an individual gains experience during development and ageing, and thus the age-related changes in information flow between the VLA and PerN are important for interpreting perceptual development and degeneration.

As Bickart *et al*. discussed in their previous study, the amygdala may play a modulatory role in the PerN^7^. In their review, Ray and Zald described the connections between prefrontal areas and the amygdala from the point of information flow direction on the basis of cytoarchitectonics^52^, but no study has used an effective connectivity technique to analyse the interaction between amygdalar subregions and associated networks. Age-related GC increase from the VLA to PerN indicates greater output signals from the VLA to PerN, a finding that is in line with the well-known conclusion that human cognition is enhanced as experience increases. In contrast, the U-shaped trend of age-related GC from PerN to VLA indicates more input signals from the perceptual cortex to the amygdala for older compared to young adults, a finding which may indicate why older adults become more sensitive. Additionally, a social psychology study reported that human social perception has an inverted U-shaped trajectory throughout life^4^. We also found that the information flow VLA → PerN showed an inverted U-shaped trend during life and negatively correlated with SRS score in ageing, results that indicate elderly social perception is degraded.

Additionally, the GC of PerN → VLA was irrelevant to the utilised behavioural scales (SRS and STAI scores) during development, but was positively correlated during ageing. This finding revealed that decreased perceptual abilities during ageing may lead to social cognitive disorders and increase social anxiety in the older adult social life. Of particular note, although both amygdalar and perceptual network activities increase with ageing, the decreased social perception might be related to a change in information flow. Consistent with previous studies, the enhanced GC from VLA to PerN, which reflects augmented information flow, indicates that the VLA upregulates the PerN. Thus, a degenerated perceptual cognition might be associated with a reduced VLA role in PerN regulation, a phenomenon that could result in older adults becoming more sensitive and susceptible to infections as well as their attenuated social response capacity. In general terms, our findings not only indicate the ability of the amygdala to use a top-down control mechanism to regulate the PerN during the lifespan, but also provide an information basis for inferring behavioural intention, sensory-motor system and high-order cognitive processes.

### Information flow between the MedA and AffN

Previous studies suggest that the MedA is involved in affiliative behaviours^53,54^. Behavioural and neuroimaging evidences illustrated that the brain network specific to prosocial behaviours require a long period for maturation, but prosocial behaviours decline with pubertal development^55^. Several functional magnetic resonance imaging (fMRI) studies reported that amygdalar activation to emotional faces and functional connectivity within the subregion that anchors in the AffN decreases from adolescence to adulthood^56^ Compared to younger adults, older adults partake in more prosocial behaviours because of empathy induction, a finding that suggests older people are likely to be more motivated to help others due to prioritisation of socioemotional goals^57^. Of note, in line with these previous findings, our U-shaped pattern for the GC of the MedA to AffN during life demonstrates that output signals from MedA to AffN are reduced from puberty to adulthood, and consequently the MedA sends more output information to the AffN with ageing. This phenomenon supports the MedA role in prosociality. Therefore, our analysis concurs with the explanation that adolescents are more easily agitated and more motivated by incentives than adults^58^. The negative correlation between the social cognitive scale and GC of the MedA → AffN indicates declined social recognition for old people, a result that demonstrates the neural analyses are consistent with human behaviours.

However, the GC of AffN → MedA and net flow of MedA → AffN were irrelevant for age based on our results. A possible explanation for this finding is that the number of participants was too small and/or the represented ages were not uniformly distributed. Another interpretation is that the factors which facilitate attachment and prosociality development are complicated. These factors are involved in peer relationships, social competence, academic achievement, caring, respect, fairness and perspective-taking skills, all of which lead to particular difference in an individual’s information input from the AffN to MedA. Consequently, our investigation on AffN during life provides a neural basis for leading adolescents to form more positive emotional incentives and targeted behavioural habits and assists older adults to maintain emotional well-being.

### Information flow between the DorA and AveN

Recent studies highlighted the amygdalar involvement in human avoidance and escape behaviours. Notably, several previous structural MRI studies offered strong evidence for early maturation of the amygdala and protracted development of AveN-relevant brain regions (ACC, pre-supplementary motor area and anterior insula)^59–61^, but the functional connectivity between ventral striatum-insula and ventral striatum-dorsal ACC gradually declines during development^58^. The stronger functional connectivity between the ventral striatum and insula reflects a higher dependence on motivational behaviours that were saliently simulated by physiological arousal in late childhood. In terms of these data, the amygdala matures fast during development, whereas the social AveN-relevant regions mature during adulthood. Our finding that the GC of the AveN → DorA increased with ageing indicates gradually elevated input signals from the AveN to DorA during life, in agreement with prior studies, and demonstrates that older individuals may exhibit more loss aversion. In contrast to young people, older adults were less negatively influenced by experienced emotional reactions via minimised emotional reactivity, a finding that suggests older adults show better emotional regulation when they employ avoidance strategies^62^. Additionally, the GC net flow from the DorA to AveN gradually decreased with age. For low negative arousal, an age-related increase in activation of the emotional control region led to decreased bilateral amygdalar activity, a result that shows older adults exhibit spontaneous engagement in the downregulation of their negative emotional responses^63^.

Taken together, our findings explored the trend of the social AveN during development and ageing to provide a theoretical basis for helping adolescents build a better AveN and assist older individuals to stay vigilant over their external environment. Moreover, our findings offer a new and comprehensive insight for neural connectivity between the amygdala and AveN during life.

## Limitations

Although this study explored the age-related relevance between the overall topological properties of the brain during the human lifespan, there were two noteworthy limitations to our investigation. First, the NKI-RS database does not have direct perception-, affiliation- or aversion- relevant scales. In this paper, we analysed perception-related SRS and negative-emotion-related STAI scores instead of using direct scales, a design which might limit result analysis. In future studies, we intend to apply direct perception-, affiliation- and aversion-relevant scales to uncover the trend of social cognition throughout the lifespan. Second, we studied GC between the amygdalar nuclei and their corresponding networks over a wide age range, but performance during infant and early childhood development is also significantly important. It is crucial that future datasets have a wider age distribution and more individuals are important to detect the neural mechanisms that underlie the networks between amygdalar nuclei and their corresponding areas.

## Conclusion

In summary, we tested alterations in the causal flow between social emotional function relevant amygdalar subregions and their associated networks across the entire lifespan. In other words, the Granger information flow between amygdalar subregions and their corresponding networks during life exhibited a long and complex trajectory. Our findings provide a new and comprehensive insight for variability of neural mechanism between social behaviours and cognition during life.

## Materials and Methods

### Participants and data acquisition

The initial fMRI and MRI data come from the Nathan Kline Institute/Rockland Sample (NKI–RS, http://fcon_1000.projects.nitrc.org/indi/pro/nki.html). One-hundred-six right-handed subjects (46 females, age range: 10–85 years, mean age: 38.8 years; 60 males, age range: 7–81 years, mean age: 38.3 years; age: <15, sample size: 11; age: 16–25, sample size: 32; age: 26–45, sample size: 24; age: 46–65, sample size: 23; age: >65, sample size: 16) were chosen from the NKI–RS database without diagnosed mental disorders or unusable anatomical images due to excessive head motion. The data were collected according to protocols approved by the institutional review board of the Nathan Kline Institute. Institutional Review Board Approval was obtained for this project at the Nathan Kline Institute (Phase I #226781 and Phase II #239708) and at Montclair State University (Phase I #000983A and Phase II #000983B). Written informed consent was obtained for all participants. Written informed consent and assent were also obtained from minor/child participants and their parents and/or legal guardian for study participation.

Structural and functional images were collected using a 3-Tesla Siemens Trio scanner. High resolution T1-weighted structural data were acquired using the magnetisation-prepared rapid gradient echo (MPRAGE) sequence with TR/TE = 2500/3.5 ms, flip angle = 8°, thickness = 1.0 mm, slices = 192, matrix = 256 × 256 and FOV = 256 mm^2^. T2-weighted resting-state functional data were acquired using a single shot, gradient-recalled echo-planar imaging (EPI) sequence with TR/TE = 2500/30 mm, flip angle = 80°, FOV = 216 mm^2^, in-plane matrix = 64 × 64, slices = 38 and thickness = 3.0 mm, for a total of 260 volumes. In order to ensure steady-state longitudinal magnetisation, the first 4 volumes were excluded.

### fMRI data preprocessing

A sequence of steps was applied to preprocess the dataset using the Statistical Parametric Mapping software based on SPM12 (http://www.fil.ion.ucl.ac.uk) and REST software (http://restfmri.net/forum/REST_V1.8). First, slice-timing correction was performed to correct each voxel’s time series during acquisition. Second, functional images were realigned for head-motion correction with rigid-body transformation (translation ≤1.5 mm and rotation ≤1.5°). Third, images of each subject are registered to the individual subjects' T1 structural image, then data were spatially normalised to the Montreal Neurological Institute (MNI) standard template, and voxels resampled to 3 × 3 × 3 mm^3^. Fourth, data were spatial smoothed using a Gaussian kernel with a 5 mm full-width and half-maximum (FWHM) to ensure a high signal-to-noise ratio (SNR). Fifth, to remove the linear low frequency drift and physiological noise, low pass filtering was performed to extract the low frequency signal range over 0.0078–0.10 Hz. Sixth, considering the impact of micro-level head motion on functional connectivity and social network patterns, we excluded sources of 24 variances via a Friston-24 model, including 6 parameters derived from the rigid-body head motion correction, 6 parameters of head motion one time point before and 12 corresponding squared items, and we also removed the mean signals over the whole brain, white matter and cerebrospinal fluid.

### Identifying amygdalar subregions

In order to identify the networks (PerN, AffN and AveN), three brain ROIs outside the amygdala were selected with MNI coordinates: lOFC (+/−38, 34, −18), vmPFC (0, 32, −12), and cACC (0, 16, 32). These ROIs interconnect with the VLA, MedA and DorA, respectively^7,64,65^.

We then generated spherical seed regions (lOFC, vmPFC and cACC), 3 mm in radius, and computed a Pearson’s product moment correlation coefficient, *r*, between the averaged time series within each seed region and the time series in each cerebral hemisphere. Next, the resultant correlation maps were converted to *z* (*r*) values using Fisher’s *r*-to-*z* transformation. The entire amygdala was displayed using Harvard–Oxford Subcortical Structural Atlas probabilistic maps, and only the voxels that had 50% or higher probability were labeled as amygdala (left: 2106 mm^3^, right: 2268 mm^3^).

Next, we carried out a one-sample *t*-test (p < 0.05, with familywise error correction [FWE]) over resultant Fisher-*z* transformed functional connectivity maps. Applying the resultant Harvard–Oxford probabilistic map of the amygdala as a mask, we selected the brain area which had statistical significance, and conducted contrast analyses on the maps from each ROI using a paired *t*-test (p < 0.05, with FWE), i.e., lOFC > vmPFC and cACC; vmPFC > cACC and lOFC; cACC > vmPFC and lOFC.

### Identifying amygdalar-subregion -based networks

We built spherical seed ROIs (VLA, MedA and DorA), 3 mm in radius, and computed the averaged time series within each amygdalar subregion. Subsequently, we used these seed ROIs to produce hemispherical functional connectivity maps. Next, we converted the resultant functional connectivity maps to *z*(*r*) values using Fisher-*z* transformation, and calculated a one sample *t*-test for transformed functional connectivity within bilateral VLA, MedA, DorA, lOFC, VMPFC and cACC (threshold p < 10^−5^ and cluster size > 10 voxels)^54^. Irrespective of the covariates with respect to age and gender, we binarised each amygdalar subregion’s significance map. Given that the intrinsic functional connectivity between amygdalar subregions and corticolimbic regions that delineate a certain social network, PerN was defined by the binary intersection maps of VLA and lOFC, AffN was defined by the binary intersection maps of MedA and vmPFC, and AveN was defined by the binary intersection maps of DorA and cACC.

### Causal interaction between amygdalar subregions and associated networks

#### Causal analysis

To investigate causal interaction between the three amygdala subregions (VLA, MedA and DorA) and their associated functional networks (PerN, AffN and AveN), GCA was proposed to estimate the effective connectivity between the reference time series of each amygdalar subregion and each voxel’s time series of its corresponding network. The time-series-relevant GCA was performed using REST-GCA in the REST toolbox (http://www.restfmri.net/forum/REST_V1.8). GCA assesses how well the current signal at a given node may be predicted from signals at previous time points in other nodes of the network, and GC is often used for fMRI data analysis via vector autoregression, as denoted by the following equations:

1 $$\begin{array}{c}{Y}_{t}=\sum _{n=1}^{m}{\alpha }_{n}{X}_{(t-n)}+\sum _{n=1}^{m}{\beta }_{n}{Y}_{(t-n)}+{\rm{C}}{Z}_{t}+{E}_{t}\\ {X}_{t}=\sum _{n=1}^{m}{\alpha ^{\prime} }_{n}{Y}_{(t-n)}+\sum _{n=1}^{m}{\beta ^{\prime} }_{n}{X}_{(t-n)}+{\rm{C}}^{\prime} {Z}_{t}+{E}_{t}^{\text{'}}\end{array}$$

where *X*_*t*_ and *Y*_*t*_ denote two time series, *a*_*n*_ and ${\alpha ^{\prime} }_{n}$ represent symbol path coefficients, *β*_*n*_ and ${\beta }_{n}^{\text{'}}$ represent autoregressive coefficients, *Z*_*t*_ denotes covariates, and *E*_*t*_ and ${E}_{t}^{\text{'}}$ represent residuals. In our study, the time series for each amygdalar subregion (VLA, MedA or DorA) was defined as time series X, and the time series of each voxel in its interconnected social network (PerN, AffN or AveN) was defined as Y. GC between each amygdalar subregion and voxels of the respective brain network was studied using bivariate coefficient GCA. Finally, we converted GCA maps for all subjects to *z*-values using Fisher’s *r*-to-*z* transformation.

### Statistical Analysis

#### One-sample t-test

Within-group analysis of GC between VLA and PerN, MedA and AffN, and DorA with AveN was conducted using a one-sample *t*-test, by which we compared the *z*-value of each voxel to a normal distribution with mean of zero and an unknown variance (p < 0.05, FDR corrected). Subsequently, we extracted mean GC values that were statistically significant and computed their Granger causal net flow.

#### Age-related GLM

Specifically, we created an age-related GLM to examine GC changes between amygdalar subregions (VLA, MedA and DorA) and their corresponding brain networks (PerN, AffN and AveN) during life. In order to investigate the casual interaction between amygdalar subregions and social networks for ageing individuals, we established multiple linear regression equations with gender as covariate and age^2^ as the predictive factor. Concretely, the GLM model can be expressed with the following equations:

2 $$\begin{array}{c}{\rm{EC}}={a}_{0}+{a}_{1}\times age+{a}_{2}\times sex\\ {\rm{EC}}={a}_{0}+{a}_{1}\times age+{a}_{2}\times ag{e}^{2}+{a}_{3}\times sex\end{array}$$

The age-relevant prediction model was generated using Akaike’s information criterion^66^, and the regression coefficients of model predictor variables were statistically analysed using a one sample *t*-test. When GC between the amygdalar subregion and network exhibited significant quadratic age effects, the peak age could be calculated using the following formula:

3 $${{\rm{Age}}}_{{\rm{peak}}}=\frac{-{a}_{1}}{2{a}_{2}}$$

#### Behavioural scale correlation analysis

To investigate the age-dependent relationship between causal interaction and emotional cognition, we examined correlation between the GC values and behavioural parameters, including SRS and STAI (p < 0.05), during life. We then plotted the fitted quadratic curve of the obtained GC values versus age, and also computed Pearson’s correlation between the GC values across the range that centered on the peak age and behavioural scales (p < 0.05).
